# 1-year trajectories of patients undergoing primary total hip arthroplasty: Patient reported outcomes and resource needs according to education level

**DOI:** 10.1186/s12891-022-05004-6

**Published:** 2022-01-25

**Authors:** Amanda I. Gonzalez, Uyen-Sa D. T. Nguyen, Patricia Franklin, Christophe Barea, Didier Hannouche, Anne Lübbeke

**Affiliations:** 1 Division of Orthopaedics and Trauma Surgery, Geneva University Hospitals, Rue Gabrielle-Perret-Gentil 4, 1205 Geneva, Switzerland; 2 Department of Biostatistics & Epidemiology, University of North Texas Health Science Center, School of Public Health, Fort Worth, USA; 3 Institute for Public Health and Medicine, Northwestern University, Evanston, USA; 4 Nuffield Department of Orthopaedics, Rheumatology and Musculoskeletal Sciences, University of Oxford, Oxford, UK

**Keywords:** Arthroplasty, Education, Patient reported outcomes

## Abstract

**Background:**

Objectives were first to evaluate by education level one-year trajectories of pain, function and general health, as well as hospital resource and medication needs in patients undergoing primary total hip arthroplasty (THA); and second, to evaluate whether outcome differences are related to existing baseline differences in health and disease severity.

**Methods:**

We included all primary THAs from a public hospital-based prospective arthroplasty registry, performed in a high-income country 2010 to 2017. Education was classified in three levels: ≤8years of schooling (low), 9-12years (medium), and ≥13years (high). Pain and function prior to and one-year after surgery were assessed with the Western Ontario McMaster Universities score (WOMAC) and general health with the 12-item short-form health survey (SF-12).

**Results:**

Overall 963 patients were included, 340 (35.3%) with low, 306 (31.8%) with medium, and 317 (32.9%) with high education. With increasing educational level preoperative scores for pain, function and SF-12 mental health component increased. One year after surgery improvement was observed in all education categories for WOMAC pain and function, SF-12 mental and physical component. However, absolute postoperative scores remained lower in all four domains for the low education group. After adjustment for baseline characteristics differences were much attenuated and no longer significant. There was also greater resource need in low educated patients.

**Conclusions:**

The inferior absolute results one year after surgery in less educated patients were largely due to older age, worse preoperative health and greater symptom severity calling for greater attention to timely and equal management, for more targeted perioperative care and increased support for the lower education group.

## Background

Total hip arthroplasty (THA) is performed to improve pain and function. Main outcomes commonly measured include revision and complication rates, mortality, and patient-reported outcomes (PRO). PROs have gained much importance in health care evaluation, health technology assessment and quality improvement, and are increasingly used in clinical studies of medical devices [1, 2]. Furthermore in 2019, the Organization for Economic Cooperation and Development (OECD) published for the first time country-specific PROs for selected conditions including joint arthroplasty [3].

Patient-related factors that influence postoperative PROs comprise patient baseline health status, including BMI, age, comorbidities, preoperative hip pain, mental health and physical function [4], as well as socioeconomic status (SES) [5].

SES is a complex construct measured by different indicators like education, employment, income, or household conditions [6–8]. Retirement or illness often change the occupation and income status, thus becoming less reliable to determine SES, while education is generally a stable indicator of SES over lifetime and unchanged by working circumstance, age or disease. In elderly patients, education is one of the most widely used indicators for SES [7]. SES affects health status throughout life [8]. For instance, better general health status [9] and lower mortality [10] are reported in elderly people with higher SES. In arthroplasty, morbidity and mortality after THA are strongly associated with SES [11], and people with low SES less often undergo primary hip replacement [12], whereas higher surgical rates of total hip and knee arthroplasty were observed with postsecondary education [13]. Education and health are positively linked by the work and economic conditions, greater social-psychological resources, and healthier lifestyle [14].

The influence of education level on patient reported outcomes (PROs) after THA has been studied in several countries. Most studies found relatively similar improvement in PROs, but lower absolute postoperative values in the lower education group [4, 15–19]. The reasons for this have received very limited attention [17, 20]. Moreover, the literature on education level and resource needs after THA is sparse. And finally, we are not aware of any study evaluating the influence of education level on PROs and resource needs after hip arthroplasty in Switzerland. Since patient population and health care access and delivery differ between countries it is important to study the country-specific situation.

The objectives of this study were first to evaluate by education level the one-year trajectories (from just before to one year after surgery) of pain, function, general health and resources needs in patients undergoing primary THA; and second, to assess whether differences in outcomes were related to existing baseline differences.

## Methods

### Study population

**Fig. 1:**
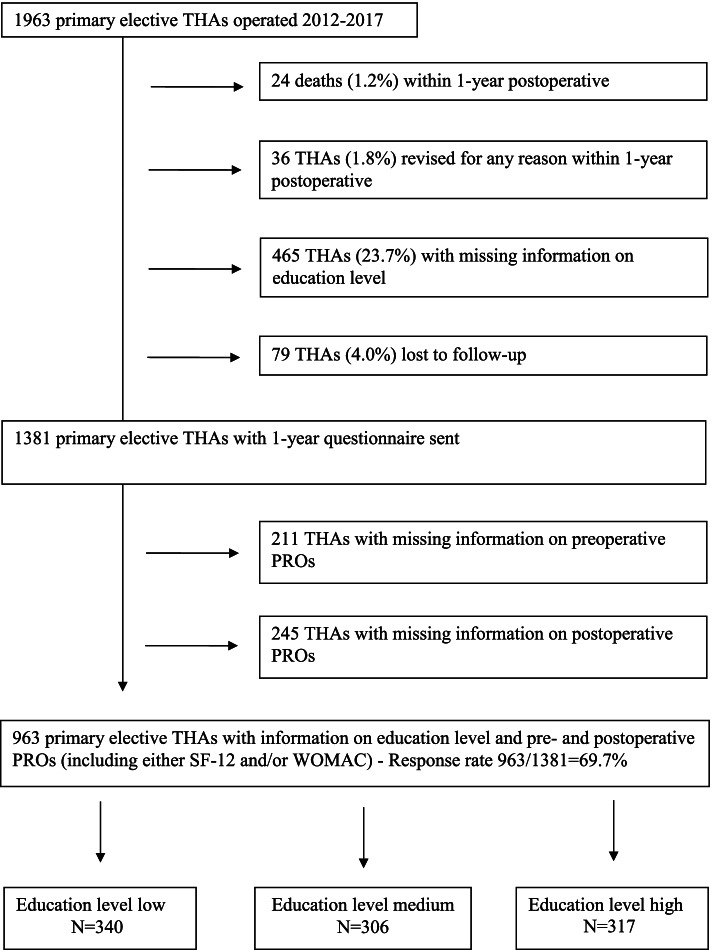
Flowchart

All primary elective THAs performed between January 2012 and May 2017 at a large University hospital in a high-income country with universal access to health care were eligible for this cohort study. Primary THAs performed for tumor or recent fracture were excluded. In case of bilateral THA, only the first hip was included. During the inclusion period 1,963 primary elective THAs were performed. Patients who were lost to follow-up, or who died within the year after surgery were excluded, as well as those with missing information on education level (Fig. 1). Overall, 1,318 patients had received the preoperative and 1-year postoperative questionnaires. Of those, the 963 patients, who had responded to both (response rate 69.7%) were included in the analysis. All data were collected prospectively as part of the hospital arthroplasty registry. Ethical approval was obtained for the hospital-based arthroplasty registry (reference no. CER: 05-017 (05-041)).

### Exposure

Education was assessed prior to surgery, and is classified in three levels: ≤8 years of schooling (low), 9-12 years (medium), and ≥13 years (high) according to the International Standard Classification of Education (ISCED) [21]. In Switzerland the years of schooling are counted as 9 years of compulsory school (primary education), then 3 to 4 years of school for high school (secondary education) and after 12 years of schooling access to bachelor/master programs.

### Outcomes and covariates

Outcomes of interest were trajectories of pain, function and general health from prior to surgery to one year after surgery as well as length of stay, discharge destination (home vs. rehabilitation), and pain medication use at discharge and 1-year after surgery (acetaminophen, nonsteroidal anti-inflammatory drugs (NSAIDs) and opioid use; any use).

Pain and function were assessed with the short-form Western Ontario McMaster Universities score (WOMAC) and general health with the 12-item short-form health survey (SF-12). The WOMAC is a self-administered 12-item questionnaire assessing pain (5-items) and function (7-items). We used the WOMAC short version by Whitehouse et al. [22]. The WOMAC score is a questionnaire developed for patients with osteoarthritis in the knee and/or hip. Values range from 0 to 100, with 0 the worst and 100 the best health status. The SF-12 is a general health status instrument. It is a self-administered 12-items questionnaire comprising two summary measures, the physical (PCS) and the mental health (MCS) component score [23]. Both the WOMAC and SF-12 have been validated in French for this study population [24, 25]. Both tests SF-12 [23] and WOMAC [22] showed good reliability

Information on pain medication use at discharge, length of stay in acute hospital, and discharge destination were obtained from the discharge summary.

The following patient-related covariates were assessed preoperatively: sex, age, body mass index (BMI), self-rated health assessed with the first question of the SF-12 (very-bad, good vs. very good/excellent), American Society of Anaesthesiology (ASA) score [26], diabetes, hypertension, cardiac disease, Charnley disability grade (A, B vs. C), smoking status, type of osteoarthritis (primary vs. secondary osteoarthritis), pain medication use (acetaminophen, NSAIDs and opioids), and insurance type (private vs. public). Secondary osteoarthritis included osteoarthritis due to dysplasia, inflammatory arthritis, post-traumatic osteoarthritis and avascular necrosis. The ASA score is a grading system used by the anesthesiologist to describe the patient’s pre-operative status (1: normal healthy patient, 2: patient with mild systemic disease, 3: patient with severe systemic disease, 4: patient with severe systemic disease that is a constant threat to life). The ASA score was analyzed as follows: grade 1 vs. 2 vs. 3 and 4 [26]. The Charnley disability grade assessed the walking capacity: group A included patients with only one hip involved, group B included patient with both hips involved but no other conditions affecting the ability to walk, and group C included patients with conditions affecting the ability to walk [27].

The following surgery-related factors were assessed: surgical approach (anterior, lateral, other), type of fixation (uncemented, hybrid, cemented) and bearing surface (ceramic-on-highly cross-linked polyethylene vs. other).

### Data collection

Information on pre-operative status and the surgical intervention was routinely documented by the operating surgeon on pre-specified data collection forms. Information on BMI and comorbidities (including ASA score, diabetes, hypertension, cardiac disease and pain medication use) was collected via the anesthesiology report. All information on patient- and operation-related factors mentioned above was collected prospectively.

Outcomes at one year after surgery were assessed via questionnaire, which were sent by mail systematically 7-10 days prior to surgery and 1 year after surgery to all patients still alive. A reminder was sent to all non-responders three to six months after the first follow-up questionnaire.

### Statistical analysis

The distribution of continuous variables and assumption of normality were assessed to determine the appropriate statistical test to use. For continuous variables, means and standard deviations were calculated. Categorical variables were expressed as proportions (%). Statistical significance was assessed using t-test for continuous variable and Pearson chi-square for categorical variables. To assess for statistical significance among group means one-way analysis of variance (ANOVA) method was used. To evaluate improvement in PROs from pre-surgery to 1-year postoperative, the gain was calculated for each education level. Comparison of the magnitude of improvement among the three education groups was performed using general linear models with high education level as reference category. To quantify the difference between the pre- and 1-year postoperative score, we calculated the effect size, Cohen’s d.

To assess whether differences in postoperative outcomes are related to existing baseline differences in health and disease severity absolute PRO results 1-year after THA were compared between the education levels using general linear models (high education=reference category). Adjustment was performed for the following variables: ASA score, type of osteoarthritis, Charnley disability grade, age, BMI, sex, self-rated health and corresponding preoperative PRO level. To evaluate resource needs linear (for length of stay) and logistic (for rehabilitation and medication need) regression analyses were performed adjusting for ASA score, type of osteoarthritis, Charnley disability grade, age, BMI, sex, self-rated health and preoperative levels of pain and function.

## Results

Overall, 963 primary THAs (mean age 68.1 ±12years, 52.8% women) were included. Of those, 340 (35.3%) were in patients with low, 306 (31.8%) with medium, and 317 (32.9%) with high education level. Preoperatively, those with low vs. high education were more often women (56.2% vs. 47%), older (71 vs. 64.8 yrs.), more obese (32.6% vs. 14.9%), had more often hypertension, diabetes, a Charnley C grade and an ASA score 3-4 (21.2% vs. 7.9%) (Table 1). Only 15% of the low education group rated their health as very good/excellent compared to 27.1% of the medium and 39.1% of the high education groups. Private insurance was rare among the low education group with 2.4% compared to 8.2% in medium and 30.6% in high education group. The three education groups did not substantially differ by surgery characteristics (Table 1).

**Table 1:** Patients’ characteristics prior to surgery and surgical characteristics by education level

Characteristics	Education level			
	Low, ***n***=340	Medium, ***n***=306	High, ***n***=317	***P*** value
**Women (%)**	191 (56.2)	168 (54.9)	149 (47.0)	0.041
**Age (yrs), mean (SD)**	71.0 (±10.9)	68 (±12.6)	64.8 (±11.7)	0.001
Mean age men	73.3 (±10.2)	70.1 (±12.8)	67.1 (±10.3)	<0.001
Mean age women	68.0 (±11.2)	65.9 (±11.9)	62.9 (±12.5)	0.001
**Age in categories (%)**				
<55	31 (9.1)	49 (16.0)	61 (19.2)	
55-64	62 (18.2)	67 (21.9)	74 (23.3)	
65-74	107 (31.5)	84 (27.5)	117 (36.9)	
≥75	140 (41.2)	106 (34.6)	65 (20.5)	<0.001
**BMI, mean (SD)**	28.0 (±4.9)	26.8 (±5.3)	25.9 (±4.4)	<0.001
**BMI in categories (%)**				
<25	103 (30.3)	115 (37.6)	145 (45.7)	
25-29.9	126 (37.1)	116 (37.9)	125 (39.4)	
30-34.9	78 (22.9)	56 (18.3)	36 (11.4)	
35-39.9	27 (7.9)	15 (4.9)	10 (3.2)	
≥40	6 (1.8)	4 (1.3)	1 (0.3)	0.001
**Self-rated health (%)**^**#**^				
Very bad-bad	72 (21.2)	46 (15.0)	32 (10.1)	
Good	216 (63.7)	177 (57.8)	161 (50.8)	
Very good-excellent	51 (15.0)	83 (27.1)	124 (39.1)	<0.001
**ASA score (%)**				
1	22 (6.5)	25 (8.2)	58 (18.3)	
2	246 (72.4)	232 (75.8)	234 (73.8)	
3-4	72 (21.2)	49 (16.0)	25 (7.9)	<0.001
**Diabetes (%)**	52 (15.3)	36 (11.8)	20 (6.3)	0.001
**HTA (%)**	189 (55.6)	162 (52.9)	121 (38.2)	<0.001
**Cardiac disease (%)**	21 (6.2)	21 (6.9)	14 (4.4)	0.401
**Charnley classification (%)**				
A	109 (32.1)	109 (35.6)	107 (33.8)	
B	56 (16.5)	62 (20.3)	87 (27.4)	
C	175 (51.5)	134 (43.8)	123 (38.8)	0.006
**Smoking status (%)**^**#**^				
Never smoker	204 (60.0)	184 (60.3)	188 (59.5)	
Ever smoker	136 (40.0)	121 (39.7)	128 (40.5)	0.977
**Type of osteoarthritis (%)**				
Primary osteoarthritis	304 (89.4)	276 (90.2)	271 (85.5)	
Secondary osteoarthritis	36 (10.6)	30 (9.8)	46 (14.5)	0.141
**Any medication use (%)**	188 (55.3)	169 (55.2)	176 (55.5)	0.997
**Type of pain medication use (%)**				
Acetaminophen	73 (21.5)	75 (24.5)	66 (20.8)	0.497
NSAIDs	119 (35.0)	122 (39.9)	143 (45.1)	0.030
Opioids	59 (17.4)	40 (13.1)	43 (13.6)	0.238
**Type of insurance (%)**				
Private	8 (2.4)	25 (8.2)	97 (30.6)	
Public	332 (97.6)	281 (91.8)	220 (69.4)	<0.001
**Surgical approach (%)**				
Anterior	302 (87.6)	268 (87.6)	275 (86.8)	
Lateral	15 (4.4)	11 (3.6)	19 (6.0)	
Other	23 (6.8)	27 (8.8)	23 (7.3)	0.551
**Implant fixation (%)**				
Uncemented	248 (72.9)	231 (75.5)	256 (80.8)	
Hybrid	85 (250)	68 (22.2)	58 (6.0)	
Cemented	7 (2.1)	7 (2.3)	3 (0.9)	0.160
**Bearing surface (%)**				
Ceramic-on-highly cross-linked polyethylene	326 (95.9)	284 (92.8)	295 (93.1)	
Other	14 (4.1)	22 (7.2)	22 (6.9)	0.184

*p value for continuous variable calculated with t-test or ANOVA test, *p* value for categorical variables calculated with Pearson chi-square

^#^One patients had missing information on self-rated health and 2 patients had missing information on smoking status

**Fig. 2:**
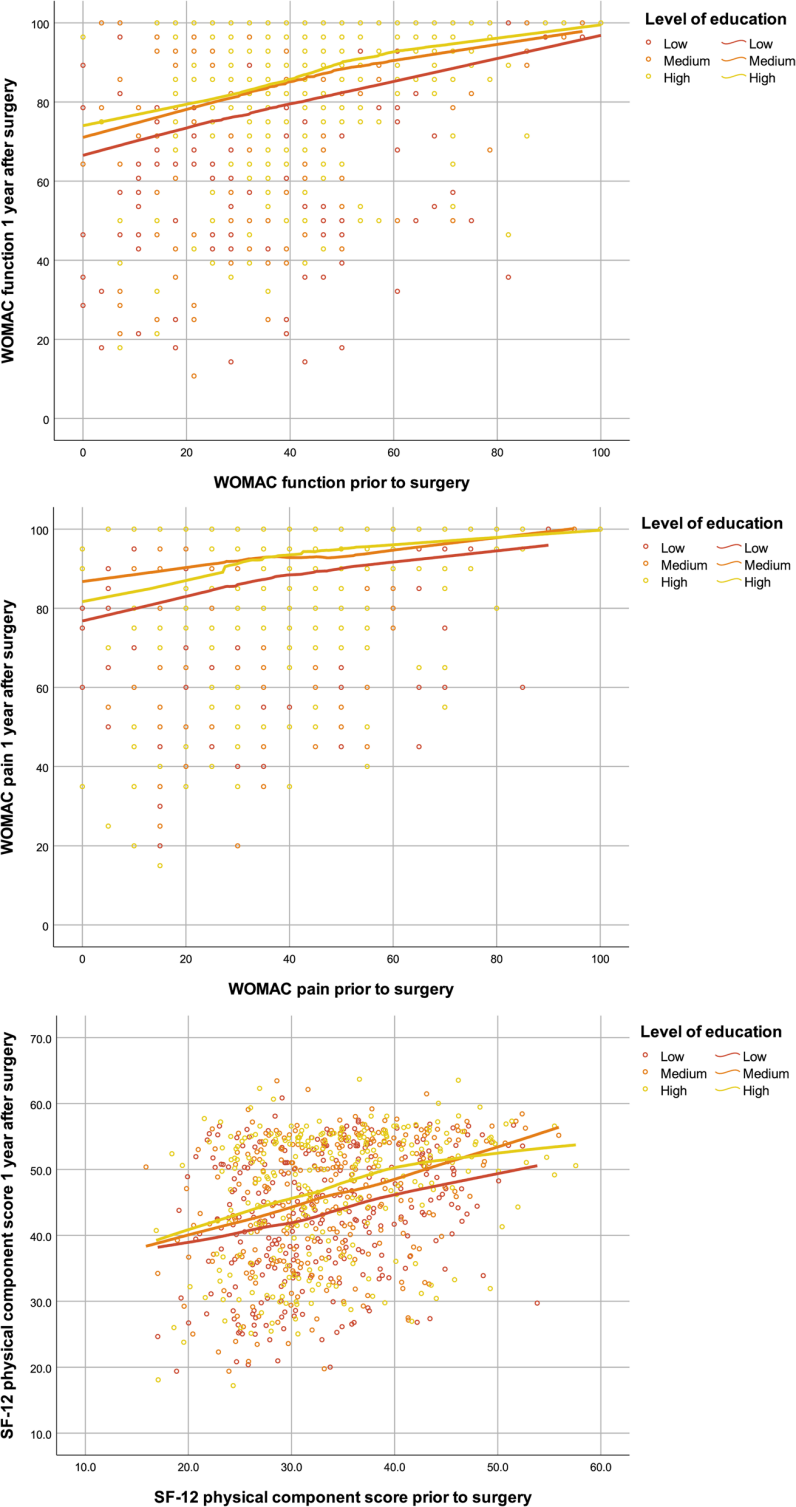
Preoperative and postoperative scores for the education groups

Pain, function and SF-12 mental component scores prior to surgery were lowest in the low education group and a gradual increase with increasing educational level was observed. SF-12 physical component scores did not differ by education (Table 2). One year after surgery, large gains in pain, function and general physical health were observed in all three groups with effect sizes between 1.2 and 2.9. Gains in mental health were also observed in all groups with effect sizes ranging from 0.2-0.5. Low education group patients had similar mean improvement in WOMAC pain and function, more improvement in the SF-12 mental component score and less in the SF-12 physical component score compared to high education group patients. However, their absolute postoperative scores remained lower in all four domains. Moreover, for any given preoperative level the postoperative score remained lower in the low education group for WOMAC pain, function and SF-12 physical function (Fig. 2). After adjustment for preoperative symptom severity and baseline characteristics, the postoperative score differences were much attenuated and no longer significant except for the SF-12 pcs (Table 3). The differences between medium and high educated were completely explained by the factors taken into account in the adjustment, whereas in the low vs. high educated comparison some difference were not explained by those factors and remained for the two WOMAC scores and the SF-12 pcs.

**Table 2:** Patient-reported outcomes (PROs) preoperative and 1-year postoperative by education level

	Education		
	Low	Medium	High
**WOMAC Pain**	*n*=340	*n*=306	*n*=317
Preoperative	36.0 (18.6)	39.2 (16.0)	43.8 (17.1)
1-year postoperative	84.3 (19.1)	87.6 (17.3)	89.6 (16.4)
Gain	48.3 (23.3)	48.4 (20.3)	45.9 (19.2)
Effect size, Cohen’s d	2.6	2.9	2.7
**WOMAC Function**	*n*=328	*n*=295	*n*=306
Preoperative	36.2 (19.1)	39.7 (17.7)	45.5 (16.9)
1-year postoperative	76.3 (22.8)	82.0 (19.7)	85.6 (17.1)
Gain	40.1 (24.8)	42.2 (22.1)	40.1 (20.0)
Effect size, Cohen’s d	1.9	2.3	2.4
**SF-12 PCS**	*n*=326	*n*=294	*n*=306
Preoperative	33.1 (7.6)	33.9 (7.4)	34.3 (7.9)
1-year postoperative	42.9 (9.3)	45.6 (9.5)	47.1 (9.5)
Gain	9.8 (10.2)	11.7 (10.0)	12.8 (10.6)
Effect size, Cohen’s d	1.2	1.4	1.5
**SF-12 MCS**	*n*=326	*n*=294	*n*=306
Preoperative	41.9 (11.5)	44.7 (10.6)	47.5 (10.3)
1-year postoperative	46.4 (11.1)	48.5 (9.8)	49.2 (10.0)
Gain	4.6 (11.4)	3.8 (10.2)	1.7 (10.3)
Effect size, Cohen’s d	0.5	0.4	0.2

**Table 3:** Patient reported outcomes (PROs) 1-year after total hip arthroplasty by education level

	Unadjusted		Adjusted^**a**^	
	β coefficient with 95% confident interval	***P*** value	β coefficient with 95% confident interval	***P*** value
**WOMAC Pain**				
Low	-5.4 (-8.1 to -2.7)	<0.001	-2.5 (-5.2 to 0.2)	0.071
Medium	-2.0 (-4.8 to 0.8)	0.154	-0.6 (-3.3 to 2.1)	0.653
High	Reference		Reference	
**WOMAC Function**				
Low	-9.0 (-12.1 to -5.9)	<0.001	-2.6 (-5.7 to 0.5)	0.098
Medium	-4.1 (-7.3 to -0.9)	0.012	-0.1 (-3.1 to 2.9)	0.950
High	Reference		Reference	
**SF-12 PCS**				
Low	-4.2 (-5.7 to -2.7)	<0.001	-1.9 (-3.3 to -0.5)	0.009
Medium	-1.6 (-3.1 to -0.1)	0.037	0.2 (-1.6 to 1.2)	0.760
High	Reference		Reference	
**SF-12 MCS**				
Low	-2.7 (-4.3 to -1.1)	0.001	-0.1 (-1.6 to 1.5)	0.949
Medium	-0.8 (-2.4 to 0.9)	0.368	0.7 (-0.8 to 2.2)	0.343
High	Reference		Reference	

^a^Adjustment performed for corresponding preoperative PRO level, sex, ASA score, type of osteoarthritis, Charnley classification, age, BMI, and self-rated health (for WOMAC Pain and Function only)

Resource need differed among the education groups. Compared to the high education group, length of stay was on average 1 day longer for the low education group (7.6 vs. 6.6 days; *p*=0.001) and discharge to a rehabilitation facility instead of their home was significantly more frequent (34.4% vs. 16.7%. *p*<0.001) (Table 4). Medication need at hospital discharge was similar among the education groups, but 1-year after THA the low education group reported more need of any medication (43.8% vs. 30.9%; *p*=0.002), in particular acetaminophen and opioids. Adjustment for age, sex, comorbidities, and preoperative symptom severity explained most of the differences observed between the low vs. high education groups. For length of stay the unadjusted odds ratio (OR) was 0.95 (95% confident interval (CI) 0.45 to 1.45), and the adjusted OR was -0.17 (95% CI -0.68 to 0.34). For rehabilitation need the unadjusted OR was 2.61 (95% CI 1.81 to 3.79), and the adjusted OR was 1.21 (95% CI 0.77 to 1.91). And for medication need 1 year after THA the unadjusted OR was 1.74 (95% CI 1.27 to 2.40), and the adjusted was OR 1.15 (95% CI 0.80 to 1.66).

**Table 4:** Hospital resources use and medication need

	Education level			
	Low ***N***=340	Medium ***N***=306	High ***N***=317	***P*** value
**Length of stay in days, mean (SD)**	7.6 (±3.5)	7.2 (±3.1)	6.6 (±3.0)	0.001
**Discharge destination (%)**				
Home	223 (65.6)	212 (69.3)	264 (83.3)	
Rehabilitation	117 (34.4)	94 (30.7)	53 (16.7)	<0.001
**Any pain medication use at discharge (%)**	315 (92.6)	280 (91.5)	293 (92.4)	0.850
**Type of pain medication use at discharge (%)**				
Acetaminophen	294 (86.5)	271 (88.6)	276 (87.1)	0.716
NSAIDs	148 (43.5)	157 (51.3)	171 (53.9)	0.21
Opioid	25 (7.4)	30 (9.8)	28 (8.8)	0.534
**Any pain medication use 1-year after THA (%)**	149 (43.8)	105 (34.3)	98 (30.9)	0.002
**Type of pain medication 1-year after THA (%)**				
Acetaminophen	102 (30.0)	75 (24.5)	59 (18.6)	0.003
NSAIDs	56 (16.5)	44 (14.4)	57 (18.0)	0.474
Opioid	31 (9.1)	12 (3.9)	13 (4.1)	0.005

*p value for continuous variable calculated with ANOVA and for categorical variables calculated with Pearson Chi-Square

## Discussion

Our study showed a large improvement in pain, function, and general health in all educational groups one year after THA. Whereas pain relief and functional improvement were similar in all groups, those with the lowest level of education improved more in mental health and less in general physical health as compared to the high education group. Despite a similar mean improvement (gain), absolute PRO levels one year after surgery were consistently lower in the low education group in all four domains (pain, function, physical and mental health). Moreover, a lower level of education was associated with a greater hospital resource need in terms of days spent in the acute care hospital and stay in a rehabilitation facility, as well as with a greater pain medication consumption one year after THA. The observed unadjusted differences in primary and secondary outcomes were substantial, and they were largely explained by greater OA symptom severity at the time of surgery, older age, worse preoperative general health and a higher comorbidity burden.

Differences in baseline characteristics between the educational groups are substantial and in accordance with previous literature [28]. In our study, both men and women with low education were on average substantially older at the time of surgery than the high and medium education groups. The difference in comorbidities at baseline is illustrated by the higher proportion of patients with obesity, hypertension, cardiac disease, diabetes, ASA score 3-4 or Charnley C grade in the lower education group. Moreover, patient-reported general health and symptom level (pain and functional impairment) before surgery also substantially differed according to education level. Similar trends have been noted by others [11, 28, 29]. This underscores the need to collect preoperative disease-specific and general PROs in evaluation of joint replacement outcomes [30]. Age, sex, and comorbidity is indeed recognized as an important variable to be included in studies comparing the outcomes of healthcare [31]. Moreover, reporting SES distribution at baseline allows assessing the diversity (or uniformity) of patients included in a study. This is particularly relevant for studies from countries without universal health care coverage, where lower SES groups are less frequently treated and as a consequence often underrepresented in studies as compared to the general population (e.g. United States) [32].

In accordance with the literature [19, 28, 33, 34] we observed an overall large improvement in PROs in the three educational groups. Greater improvement in function and general physical health but smaller gain in mental health in the higher education group was reported by others [28, 33], this is consistent with our results. Regarding the absolute PROs after surgery, most published studies observed higher values (to a variable extent) in patients with a higher education level, which was associated with better postoperative outcomes at 6 months and 3 years [4, 16, 18], consistent with our results. In another cohort study including 11,464 primary THAs [17], a higher level of education was related to slightly more favorable postoperative outcomes one year after surgery. In contrast to the before-mentioned studies, Dailiana et al. [15] reported that low education was not a predictor of poor outcome one year after total joint arthroplasty. Finally, a systematic review [35], including three studies evaluating education level and outcomes after THA [4, 18, 19] concluded that education level showed a weak evidence for a short- or long-term association with outcomes after THA.

After taking into account age at surgery and baseline status regarding osteoarthritis symptom severity, general health, medical and orthopedic comorbidities, the substantial differences in unadjusted postoperative scores observed between the education groups were largely attenuated in our study. They entirely explained the differences between medium and high educated and a substantial part but not all of the differences between low and high educated. Neuburger et al. [20] performed a similar analysis before and after total hip and knee arthroplasty, albeit using the deprivation index as measure of SES. They found that the existing baseline differences in health and disease severity explained the postoperative difference only partly - and to a lesser degree than in our study. The remaining clinically and statistically significant difference in 1-year outcome was attributed by the authors to lesser improvement in the more deprived patients. It should be noted that their measure of SES is different from ours. This is illustrated by the fact that those with the lowest SES (deprivation index in their study) were younger and more often men, whereas there were women and patients of older age in our study using education as SES measure.

We observed a longer stay in the acute care hospital, a greater need for rehabilitation care and a higher use of (weak) pain medication one year after surgery in the low education group, which was explained by older age and related higher prevalence of comorbidities among them. A longer hospital stay following total hip arthroplasty for patients with lower SES has been reported by others [36, 37]. Discharge destination has also been related to SES [38]. Moreover, Singh et al. [39] in a recent study including over 4 million patients evaluated the association of insurance status with outcomes after primary total hip arthroplasty and reported a lower hazard ratio for discharge to rehabilitation/inpatient facility for patients with higher income compared to lower income. Knowing the risk factors for poor outcomes and greater resource need, can improve preparation for the intervention and the rehabilitation period. Thus, acknowledging that patients differ at baseline according to their educational status can help both patients and care providers to anticipate and adapt support for patients following THA. A recent study evaluated predictors of recovery after THA - measured with the WOMAC - in the univariate analysis higher education level was a significant predictor, but no longer in the multiple linear regression analysis, while age, baseline symptoms, comorbidities, self-efficacy and social support remained all significantly associated with recovery [40]. Weiss et al. found an increased risk for early mortality and readmission in patients with lower SES, which was much attenuated after adjusting for age, sex and comorbidity [11].

Duration of symptoms before undergoing hip surgery tends to be longer in patients with lower socioeconomic status [41]. Information on duration of hip symptoms had not been collected in our study, but our low education group was older and had worse preoperative scores. In addition, the proportion of women in the low education group was higher. Underuse in total joint arthroplasty was reported in severe OA and gender difference in severe OA was also described, with a three times greater underuse of total joint arthroplasty in women compared to men [42]. This suggests that the time with symptoms, which depends on patients’ access to health care and indication for surgery, as well as preference for surgery, referral patterns, physician management and/or waiting time until surgery, may have been longer for this group despite universal access to health care in Switzerland. Indeed, Hawker et al. [43] also found that less education and lower income - even in a universal health care access environment - were independently associated with worse symptoms and disability. However, the willingness to undergo surgery did not differ by SES. They concluded that these disparities may be related to differences in physician management of hip arthritis and referral/indication for surgery, according to the patient’s socioeconomic situation. Thus, general practitioners and surgeons should be particularly attentive when patients with low education status report hip OA symptoms. Chronic pain affects mental health status [44], which in turn influences outcomes after THA. Reaching this population earlier might allow better management and timing of surgery, so that the lower education group could attain absolute post-surgery PROs close to those in the high education group, and their resource need could be reduced as well. Postponing the surgery does not seem to convey any advantage in terms of improvement, but might rather increase the risk of a higher comorbidity burden and associated complication rates and resource needs.

### Limitations

Due to missing data on PROs, 30% of the patients had to be excluded from the final analysis. Overall 79 patients (4%) were lost to follow-up because we couldn’t reach them (moved country or canton without a new address available to us ) or because they did not answer the questionnaire (including the reminder). However, the response rate for having returned both the pre- and postoperative questionnaire was 70%, which is well above the recommended threshold of 60% for acceptable frequency of response [5]. Furthermore, since education level was patient-reported some misclassification of the exposure remains possible. Finally, to be accurate, PROs need to be comprehensive for all patients regardless of their education level. A recently published study [45] reported that the vast majority of commonly used PROs in orthopedics are below the 6^th^ to 8^th^ grade reading level, which is the reading level recommended [46, 47].

## Conclusion

A large improvement in pain, function and general health was seen in all education groups one year after surgery in this cohort study. The inferior absolute PRO results one year after surgery and the greater resource need in less educated patients were largely due to older age at surgery, worse preoperative health and greater symptom severity calling for greater attention to timely and equal management of disease symptoms, for more targeted perioperative care and increased support for the lower education group. Moreover, including SES measures in clinical studies is important given the reported differences in pre-surgery status, in need of health care resources and in postoperative patient-reported outcomes.

## Data Availability

The Geneva Arthroplasty Registry obtains patient consent for data collection and protects access to the data. We have established data use procedures through our Publication and Ancillary Studies Committee. Investigators can formally request analytic access to the data through these mechanisms. This work was performed at the Division of Orthopaedics and Trauma Surgery, Geneva University Hospitals, Geneva, Switzerland.

## Abbreviations

THA: Total hip arthroplasty
PRO: Patient-reported outcomes
OECD: Organization for Economic Cooperation and Development
SES: Socioeconomic status
ISCED: International Standard Classification of Education
NSAIDS: Nonsteroidal anti-inflammatory drugs
WOMAC: Western Ontario McMaster Universities score
SF: Short form
PCS: Physical component score
MCS: Mental health component score
BMI: Body mass index
ASA: American Society of Anaesthesiology
OR: Odds ratio
CI: Confident interval
OA: Osteoarthritis

## References

[1] Mercieca-Bebber R, King MT, Calvert MJ, Stockler MR, Friedlander M (2018). The importance of patient-reported outcomes in clinical trials and strategies for future optimization. Patient Relat Outcome Meas.

[2] Weszl M, Rencz F, Brodszky V (2019). Is the trend of increasing use of patient-reported outcome measures in medical device studies the sign of shift towards value-based purchasing in Europe?. Eur J Health Econ.

[3] http://www.oecd.org/health/health-systems/Measuring-what-matters-the-Patient-Reported-Indicator-Surveys.pdf. Accessed January 12, 2021.

[4] Bischoff-Ferrari HA, Lingard EA, Losina E, Baron JA, Roos EM, Phillips CB (2004). Psychosocial and geriatric correlates of functional status after total hip replacement. Arthritis Rheum.

[5] Rolfson O, Bohm E, Franklin P, Lyman S, Denissen G, Dawson J (2016). Patient-reported outcome measures in arthroplasty registries Report of the Patient-Reported Outcome Measures Working Group of the International Society of Arthroplasty Registries Part II. Recommendations for selection, administration, and analysis. Acta Orthop.

[6] Galobardes B, Smith GD, Lynch JW (2006). Systematic review of the influence of childhood socioeconomic circumstances on risk for cardiovascular disease in adulthood. Ann Epidemiol.

[7] Shavers VL (2007). Measurement of socioeconomic status in health disparities research. J Natl Med Assoc.

[8] Winters-Miner LA, Bolding PS, JM HI, Goldstein M, Hill T, Nisbet R (2015). Practical Predictive Analytics and Decisioning Systems for Medicine Informatics Accuracy and Cost-Effectiveness for Healthcare Administration and Delivery Including Medical Research.

[9] Hemingway H, Nicholson A, Stafford M, Roberts R, Marmot M (1997). The impact of socioeconomic status on health functioning as assessed by the SF-36 questionnaire: the Whitehall II Study. Am J Public Health.

[10] Bassuk SS, Berkman LF, Amick BC (2002). Socioeconomic status and mortality among the elderly: findings from four US communities. Am J Epidemiol.

[11] Weiss RJ, Karrholm J, Rolfson O, Hailer NP (2019). Increased early mortality and morbidity after total hip arthroplasty in patients with socioeconomic disadvantage: a report from the Swedish Hip Arthroplasty Register. Acta Orthop.

[12] Dixon T, Shaw M, Ebrahim S, Dieppe P (2004). Trends in hip and knee joint replacement: socioeconomic inequalities and projections of need. Ann Rheum Dis.

[13] Mota RE, Tarricone R, Ciani O, Bridges JF, Drummond M (2012). Determinants of demand for total hip and knee arthroplasty: a systematic literature review. BMC Health Serv Res.

[14] Ross CE, Wu C (1995). The Links Between Education and Health. Am Sociol Rev.

[15] Dailiana ZH, Papakostidou I, Varitimidis S, Liaropoulos L, Zintzaras E, Karachalios T (2015). Patient-reported quality of life after primary major joint arthroplasty: a prospective comparison of hip and knee arthroplasty. BMC Musculoskelet Disord.

[16] Fortin PR, Clarke AE, Joseph L, Liang MH, Tanzer M, Ferland D (1999). Outcomes of total hip and knee replacement: preoperative functional status predicts outcomes at six months after surgery. Arthritis Rheum.

[17] Greene ME, Rolfson O, Nemes S, Gordon M, Malchau H, Garellick G (2014). Education attainment is associated with patient-reported outcomes: findings from the Swedish Hip Arthroplasty Register. Clin Orthop Relat Res.

[18] Mahomed NN, Liang MH, Cook EF, Daltroy LH, Fortin PR, Fossel AH (2002). The importance of patient expectations in predicting functional outcomes after total joint arthroplasty. J Rheumatol.

[19] Schafer T, Krummenauer F, Mettelsiefen J, Kirschner S, Gunther KP (2010). Social, educational, and occupational predictors of total hip replacement outcome. Osteoarthr Cartil.

[20] Neuburger J, Hutchings A, Black N, van der Meulen JH (2013). Socioeconomic differences in patient-reported outcomes after a hip or knee replacement in the English National Health Service. J Public Health (Oxf).

[21] Website. International Standard Classification of Education (ISCED). Available at https://read.oecd-ilibrary.org/education/isced-2011-operational-manual_9789264228368-en#page1. Accessed March 19. 2020.

[22] Whitehouse SL, Lingard EA, Katz JN, Learmonth ID (2003). Development and testing of a reduced WOMAC function scale. J Bone Joint Surg (Br).

[23] Ware J, Kosinski M, Keller SD (1996). A 12-Item Short-Form Health Survey: construction of scales and preliminary tests of reliability and validity. Med Care.

[24] American College of Rheumatology. http://www.rheumatology.org/I-Am-A/Rheumatologist/Research/Clinician-Researchers/Western-Ontario-McMaster-Universities-Osteoarthritis-Index-WOMAC.

[25] Dauphinee SW, Gauthier L, Gandek B, Magnan L, Pierre U (1997). Readying a US measure of health status, the SF-36, for use in Canada. Clin Invest Med.

[26] American Society of Anesthesiologists (1963). New classification of physical status. Anesthesiology.

[27] Charnley J (1972). The long-term results of low-friction arthroplasty of the hip performed as a primary intervention. J Bone Joint Surg (Br).

[28] Keurentjes JC, Blane D, Bartley M, Keurentjes JJ, Fiocco M, Nelissen RG (2013). Socio-economic position has no effect on improvement in health-related quality of life and patient satisfaction in total hip and knee replacement: a cohort study. PLoS One.

[29] Clement ND, Muzammil A, Macdonald D, Howie CR, Biant LC (2011). Socioeconomic status affects the early outcome of total hip replacement. J Bone Joint Surg (Br).

[30] Rolfson O, Karrholm J, Dahlberg LE, Garellick G (2011). Patient-reported outcomes in the Swedish Hip Arthroplasty Register: results of a nationwide prospective observational study. J Bone Joint Surg (Br).

[31] Grosse Frie K, van der Meulen J, Black N (2012). Single item on patients’ satisfaction with condition provided additional insight into impact of surgery. J Clin Epidemiol.

[32] Franklin PD, Miozzari H, Christofilopoulos P, Hoffmeyer P, Ayers DC, Lubbeke A (2017). Important patient characteristics differ prior to total knee arthroplasty and total hip arthroplasty between Switzerland and the United States. BMC Musculoskelet Disord.

[33] Dowsey MM, Nikpour M, Choong PF (2014). Outcomes following large joint arthroplasty: does socio-economic status matter?. BMC Musculoskelet Disord.

[34] Judge A, Cooper C, Williams S, Dreinhoefer K, Dieppe P (2010). Patient-reported outcomes one year after primary hip replacement in a European Collaborative Cohort. Arthritis Care Res.

[35] Buirs LD, Van Beers LW, Scholtes VA, Pastoors T, Sprague S, Poolman RW (2016). Predictors of physical functioning after total hip arthroplasty: a systematic review. BMJ Open.

[36] Hollowell J, Grocott MP, Hardy R, Haddad FS, Mythen MG, Raine R (2010). Major elective joint replacement surgery: socioeconomic variations in surgical risk, postoperative morbidity and length of stay. J Eval Clin Pract.

[37] Shau D, Shenvi N, Easley K, Smith M, Bradbury T, Guild G (2018). Medicaid Payer Status Is Associated with Increased 90-Day Morbidity and Resource Utilization Following Primary Total Hip Arthroplasty: A Propensity-Score-Matched Analysis. J Bone Joint Surg Am.

[38] Inneh IA, Clair AJ, Slover JD, Iorio R (2016). Disparities in Discharge Destination After Lower Extremity Joint Arthroplasty: Analysis of 7924 Patients in an Urban Setting. J Arthroplast.

[39] Singh JA, Cleveland JD (2018). Medicaid or Medicare insurance payer status and household income are associated with outcomes after primary total hip arthroplasty. Clin Rheumatol.

[40] Brembo EA, Kapstad H, Van Dulmen S, Eide H (2017). Role of self-efficacy and social support in short-term recovery after total hip replacement: a prospective cohort study. Health Qual Life Outcomes.

[41] Neuburger J, Hutchings A, Allwood D, Black N, van der Meulen JH (2012). Sociodemographic differences in the severity and duration of disease amongst patients undergoing hip or knee replacement surgery. J Public Health (Oxf).

[42] Hawker GA, Wright JG, Coyte PC, Williams JI, Harvey B, Glazier R (2000). Differences between men and women in the rate of use of hip and knee arthroplasty. N Engl J Med.

[43] Hawker GA, Wright JG, Glazier RH, Coyte PC, Harvey B, Williams JI (2002). The effect of education and income on need and willingness to undergo total joint arthroplasty. Arthritis Rheum.

[44] Hawker GA, Gignac MA, Badley E, Davis AM, French MR, Li Y (2011). A longitudinal study to explain the pain-depression link in older adults with osteoarthritis. Arthritis Care Res.

[45] Perez JL, Mosher ZA, Watson SL, Sheppard ED, Brabston EW, McGwin G (2017). Readability of Orthopaedic Patient-reported Outcome Measures: Is There a Fundamental Failure to Communicate?. Clin Orthop Relat Res.

[46] Advancing Effective Communication, Cultural Competence, and Patient- and Family-Centered Care: A Roadmap for Hospitals. Oakbrook Terrace: The Joint Commission; 2010.

[47] Weiss BD. Help patients understand: A manual for clinicians. 2nd ed. Chicago: American Medical Association Foundation and American Medical Association; 2007.

